# Anti-Inflammatory Activity of a Medicinal Herb Extract Mixture, HM-V, on an Animal Model of DNCB-Induced Chronic Skin Inflammation

**DOI:** 10.3390/plants10081546

**Published:** 2021-07-28

**Authors:** Sungbae Park, Sangmin Lee, Youngho Weon, Taewook Kim, Hakwon Kim, Taehoon Lee

**Affiliations:** 1 Hanulmaum Oriental Medicine Clinic, Hanulmaum Oriental Medicine Laboratory, Seoul 06640, Korea; mu12345@empas.com (S.P.); yhweon2@hanmail.net (Y.W.); next10years@nate.com (T.K.); 2 Department of Applied Chemistry, Global Center for Pharmaceutical Ingredient Materials, Kyung Hee University, Yongin 17104, Korea; dna1710@hanmail.net

**Keywords:** chronic skin inflammation, medicinal herb extract, chemokine, contact dermatitis

## Abstract

Chronic inflammatory skin diseases, such as atopic dermatitis, are caused by the accumulation of immune cells and the overproduction of chemokines, including CCL17 and CCL22, due to the activation of pro-inflammatory cytokines secreted from keratinocytes. In the present study, the inhibitory activity of HM-V on tumor necrosis factor alpha (TNF-α)/interferon gamma (IFN-γ)-induced pro-inflammatory cytokines was examined in human keratinocytes (HaCaTs) and 2,4-dinitrofluorobenzene (DNCB)-induced chronic skin contact dermatitis animal models. Traditional Asian medicinal herb extracts mixture (HM-V), which have been extensively used in Asian medicine, were utilized. In TNF-α/IFN-γ-induced HaCaTs, HM-V strongly inhibited mRNA and protein expression of CCL17 and CCL22 in a concentration-dependent manner. The expression of pro-inflammatory cytokines such as TNF-α, IL-1β, and IL-6 was also inhibited. Therefore, localized administration of HM-V in the DNCB-induced animal model alleviated immune cell deposition and skin inflammation. The results indicate that HM-V exerts inhibitory effects on keratinocyte production of CCL17 and CCL22. Furthermore, HM-V may be a useful anti-inflammatory agent for the prevention and treatment of inflammatory skin diseases.

## 1. Introduction

Skin blocks external stimuli and acts as the first line of defense in the human immune system [1,2,3]. The skin can experience dysfunction if subjected to sustained stimulation or immune cell infiltration due to overexpression of various chemokines and pro-inflammatory cytokines [2]. Chronic skin inflammatory disease is caused by continued immune cell activation. Inflammation in the skin can cause severe skin inflammatory diseases, such as atopic dermatitis, seborrheic dermatitis, and psoriasis [3]. In chronic skin diseases, the predominant role of pro-inflammatory cytokines, such as tumor necrosis factor alpha (TNF-α) and interferon gamma (IFN-γ) is to mediate the immune balance of surrounding skin tissues. These cytokines also promote the secretion of small signaling proteins such as chemokines [4,5]. Chemokines are small signal transduction cytokines that play an important role in immune cell deposition and mobilization. The main function of these chemokines is to recruit leukocytes, monocytes, and neutrophils into inflammatory sites [6]. In recent studies, increased levels of CC-chemokines, such as thymus, activation-regulated chemokine (TARC/CCL17), and CC-chemokine macrophage-derived chemokine (MDC/CCL22) were observed in the skin of patients with chronic skin inflammatory diseases, including atopic dermatitis. In addition, the chemokines can induce thymic stromal lymphopoietin (TSLP) expression, leading to sustained activation of Th2 cytokines in immune cells which can worsen chronic dermatitis [7,8,9]. Chemokines are divided into four subfamilies, based on cysteine residues. CCL17 and CCL22 are C-C chemokines that signal through the specific CC chemokine receptor 4 (CCR4). CCL17 and CCL22 have been reported closely associated with Th2 cell-mediated inflammatory diseases such as atopic dermatitis [10]. Both CCL17 and CCL22 share high homology (32% amino acid identity) [11]. In previous studies, CCL17 and CCL22 were reported useful as new biomarkers in patients with chronic dermatitis [12]. These chemokines have been detected at high levels in the blood of atopic dermatitis patients and were overexpressed in skin lesions [13]. Also, CCL17 and CCL22 were shown to participate in the accumulation and activation of Th2 cells. These chemokines exacerbate the chronic skin inflammation that occurs in skin diseases like atopic dermatitis, leading to persistent immune imbalances [11,12,13].

Numerous medicinal herb extracts have been used in traditional Asian medicine to treat skin disorders including measles, chicken pox, hepatitis, and skin cancer [14,15,16]. In the present study, an Asian traditional herb extracts mixture, HM-V, was prepared based on a Korean medicine recipe. HM-V components have various natural herb extracts. For instance, extracts from the *Chelidonium majus* var. *asiaticum*, Heartleaf Houttuynia, restored the immunosuppression induced by cyclophosphamide, an anti-tumor agent. Chelidonine is an important benzophenanthridine alkaloid and the main active ingredient in *Chelidonium majus* var. *asiaticum* [17,18]. In this study, HM-V suppressed TNF-α/IFN-r-induced production of pro-inflammatory cytokines and chemokines including CCL17 and CCL22 in human keratinocytes (HaCaTs). Therefore, the effects of HM-V on the production of CCL17 in CCL22 2,4-dinitrofluorobenzene (DNCB)-induced chronic skin contact dermatitis in an animal model were evaluated. The topical administration of HM-V reduced epidermal thickness, eosinophil infiltration as well as lymph node and spleen size. In addition, HM-V exerted inhibitory effects on the production of CCL17 and CCL22 in the DNCB-induced mouse model. HM-V could potentially control skin inflammation in mouse models of chronic skin inflammation.

## 2. Results

### 2.1. HM-V Inhibited TNF-α/IFN-γ-Induced Expression of Cytokines and Chemokines in HaCaTs

**Figure 1:**
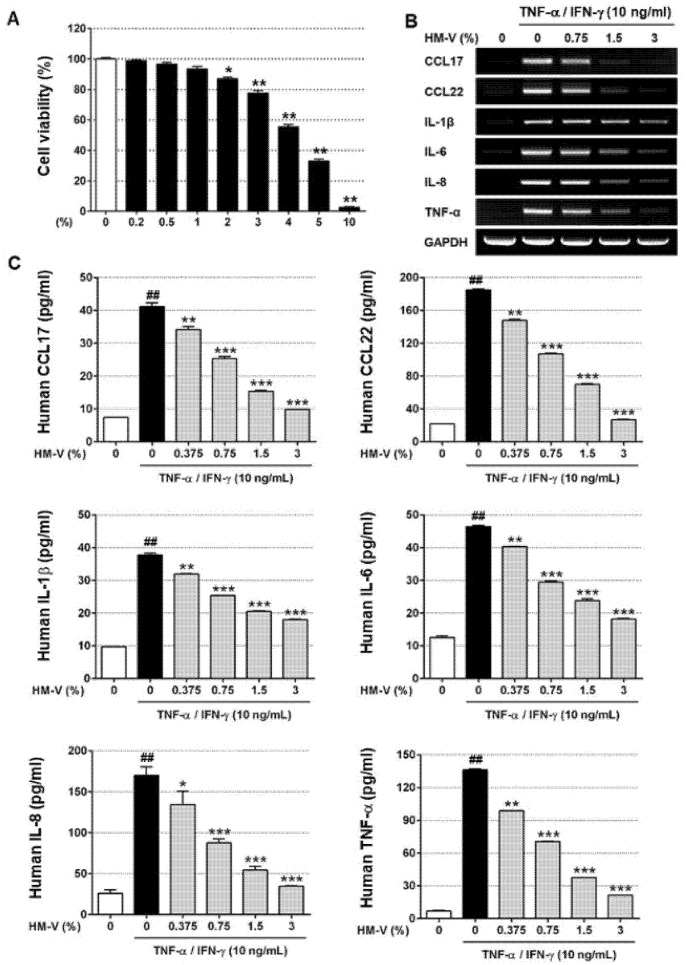
Effects of HM-V on pro-inflammatory cytokine and chemokine mRNA and protein expression in TNF-α/IFN-γ-induced HaCaTs. (**A**) Cell viability test was performed up to a concentration of 10% HM-V. The survival rate of the cells was confirmed using MTT assay and the results measured based on OD value. The viability tests were performed in triplicate and repeated at least three times. * p < 0.05 and ** p < 0.01 compared with negative control. (**B**) RT-PCR assay was performed to compare pro-inflammatory cytokine and chemokine mRNA levels. (**C**) Quantitative ELISA was performed to compare the pro-inflammatory cytokine and chemokine expression levels. The protein amounts were compared and data presented as a graph. The protein amounts represent the means ± SD from three independent experiments. ## *p* < 0.05 *versus* negative control, * p < 0.05, ** p < 0.01, and *** p < 0.001 compared with treatment with positive control.

First, the effects of HM-V on cell viability were evaluated. As shown in Figure 1, HaCaTs were treated with the indicated concentrations of HM-V. Approximately 80% of the cells treated with 3% HM-V survived for over 24 h. Therefore, up to 3% HM-V was used for the in vitro experiments. Next, the mRNA expression levels of the pro-inflammatory cytokines (IL-6, IL-1β, IL-8, TNF-α, CCL17, and CCL22) in the TNF-α/IFN-γ-induced HaCaTs were measured to evaluate the effects of HM-V. At a concentration of 3%, HM-V strongly inhibited the mRNA expression of various TNF-α/IFN-γ-induced pro-inflammatory cytokines in a concentration-dependent manner. However, IL-1β mRNA showed slightly weaker expression than other mRNAs. Next, the effects of HM-V on pro-inflammatory cytokine production in TNF-α/IFN-γ-stimulated keratinocytes were investigated. The production of pro-inflammatory cytokines was compared using ELISA. Stimulation with TNF-α/IFN-γ led to an increase of 40–180 pg/mL depending on the type of cytokine. The basal control levels were approximately 7–40 pg/mL.

### 2.2. HM-V Attenuated DNCB-Induced Skin Inflammation

**Figure 2:**
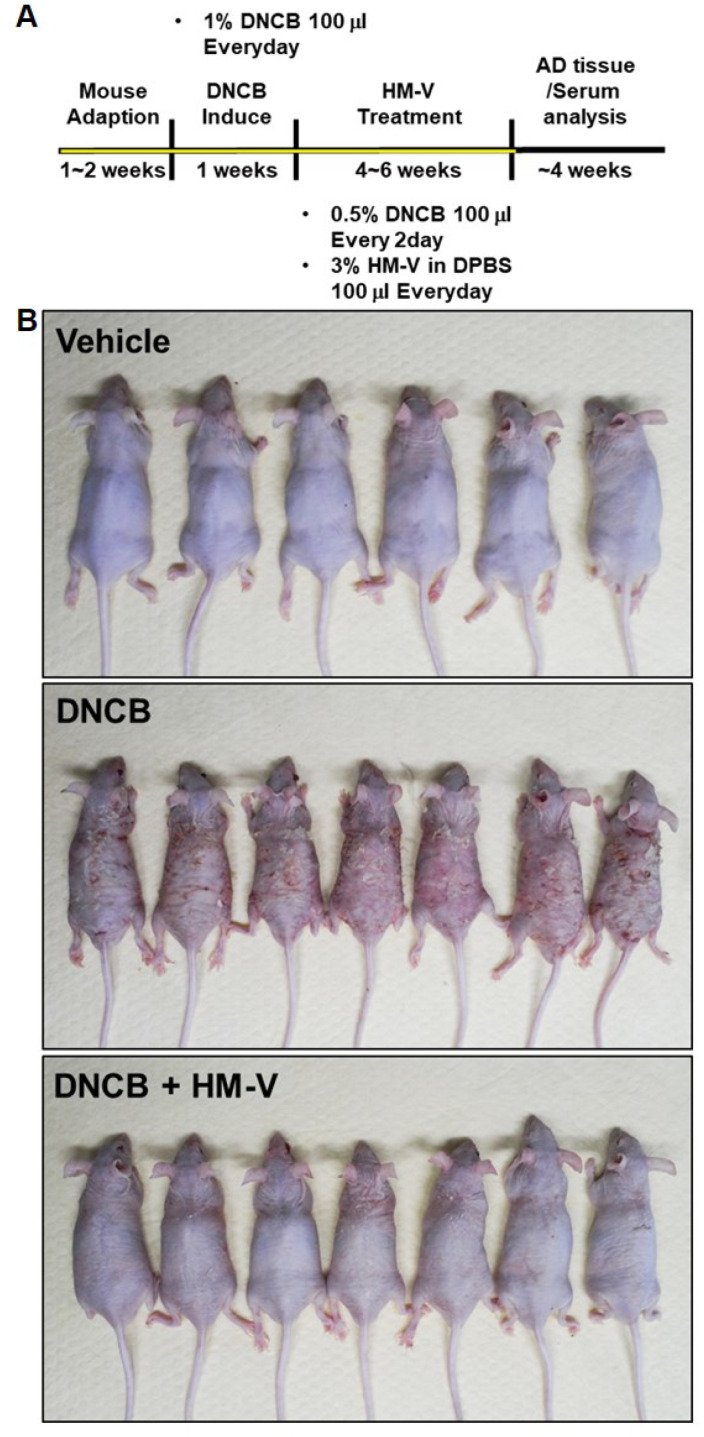
Effects of HM-V on DNCB-induced contact skin inflammation severity. (**A**) Animal experimental scheme. (**B**) Clinical severity of inflammation skin lesion. Photographs were taken on the day before mice were euthanized.

The efficacy of HM-V on atopic dermatitis-like lesions in vivo were confirmed in a DNCB-induced atopic dermatitis-like contact chronic skin inflammation mouse model. Edema, erythema, and scarring were compared between the positive control group treated with DNCB allergens and the vehicle group treated without DNCB in BALB/c nude mice. Notably, the skin inflammatory lesions caused by DNCB were clearly reduced in the test group treated with 3% topical HM-V (Figure 2).

**Figure 3:**
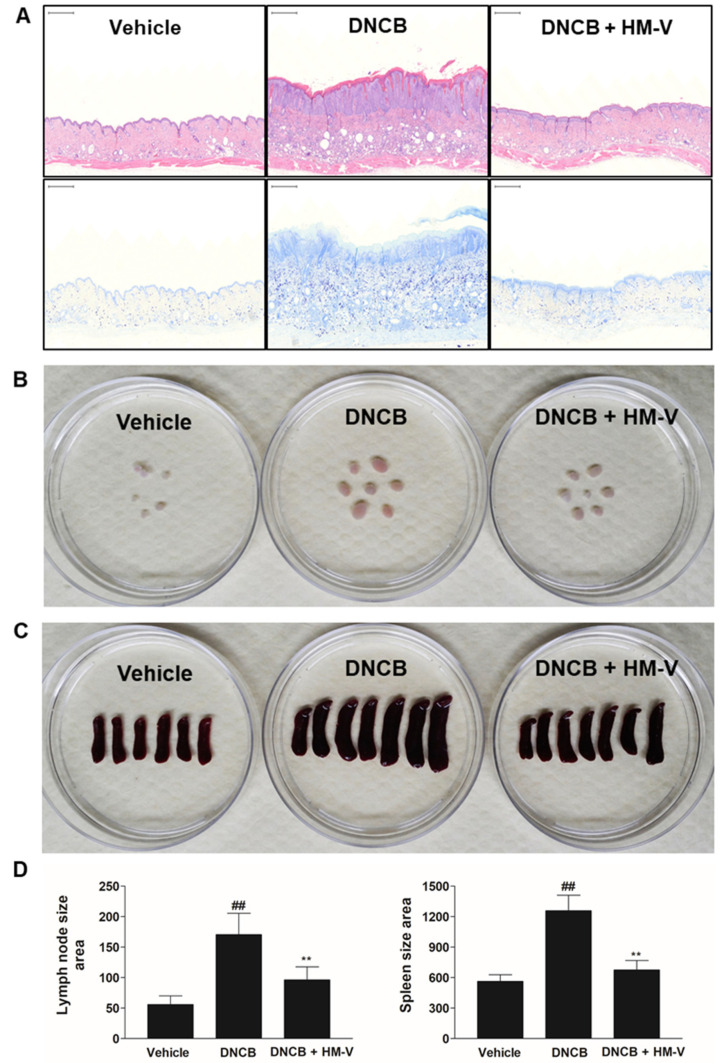
Effects of HM-V on DNCB-induced mast cell infiltration and organ size. (**A**) Mouse back skin lesions were fixed in 10% formaldehyde, sectioned into 4.5-μm slices, and stained with H&E and Toluidine Blue O. (**B**) Local lymph node size. (**C**) Spleen size. (**D**) Image analysis of lymph node and spleen. Data shown are the average of six or seven samples per group and analyzed using the ImageJ program 1.8.0_172. ## *p* < 0.05 *versus* negative control, ** *p* < 0.05 *versus* positive control.

Symptoms of atopic dermatitis are often accompanied by enlarged local lymph nodes. The spleen also increases in size due to cell-mediated reactions, including both T and B cell activation [19]. Therefore, the effects of HM-V were confirmed using morphological analysis of epidermal thickness, local lymph nodes, and spleen (Figure 3). The size of local lymph nodes and spleen increased in the positive control group treated with DNCB compared with the negative control group without DNCB. In addition, the size of local lymph nodes and spleen were significantly reduced in the test group treated with 3% HM-V compared with the positive group. DNCB induces keratosis, which is caused by the abnormal differentiation of skin tissue due to inflammation. Keratosis results in abnormal proliferation of the epidermis. The present study results demonstrated a substantial reduction in the epidermis thickness in the HM-V-treated group. Furthermore, 3% HM-V inhibited eosinophil and mast cell infiltration based on H&E and Toluidine Blue O staining assays.

### 2.3. HM-V Reduced Serum Levels of Pro-Inflammatory Cytokines and Chemokines

**Figure 4:**
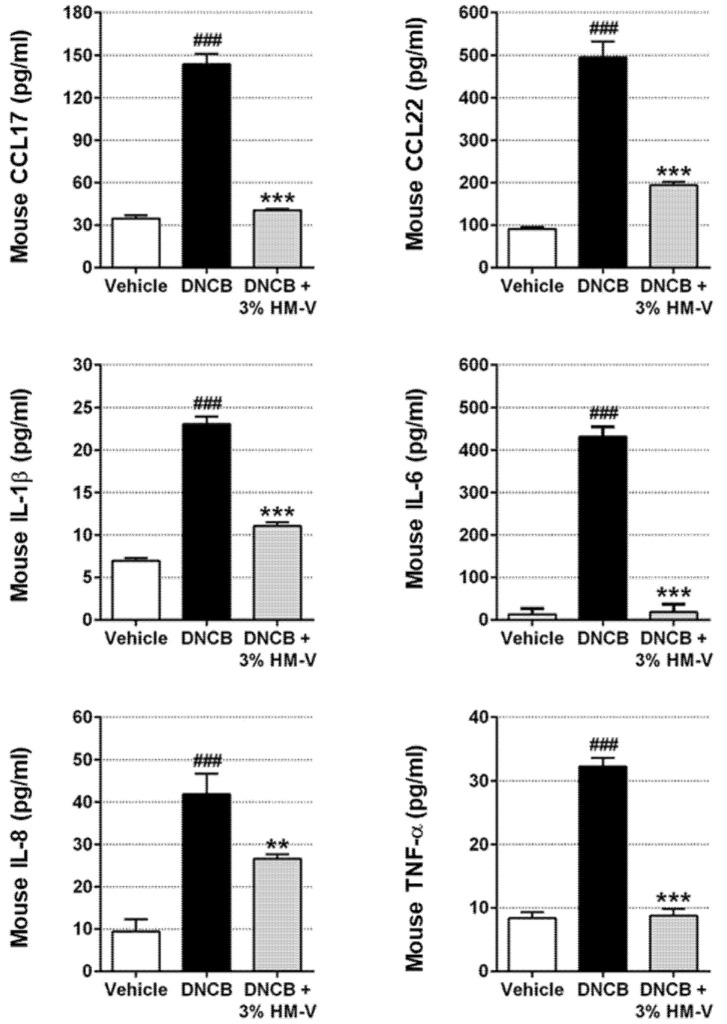
Effects of HM-V on serum pro-inflammatory cytokines and chemokines in DNCB-induced mice. Serum was collected 24 h after final HM-V and/or DNCB sensitization. Pro-inflammatory cytokines and chemokines were analyzed using ELISA. Vehicle, negative control; DNCB, DNCB-induced mice (positive control); DNCB+3% HM-V, DNCB plus 3% HM-V. Data shown are the average of six or seven samples per group and shown as the means ± SD. ### *p* < 0.001 *versus* negative control, ** p < 0.01, and *** *p* < 0.001 *versus* positive control.

Abnormal activation of CCL17, CCL22, and pro-inflammatory cytokines in HaCaTs leads to T-cell or leukocyte infiltration into the epidermis [20]. Therefore, serum analysis was performed following treatment with 3% HM-V in DNCB-induced mice. Blood was isolated from the mice as described in the experimental method section and ELISA was performed. Production of CCL17 and CCL22 increased to 140.1 ± 14.35 pg/mL and 495 ± 11.97 pg/mL from basal levels of 37.016 ± 4.263 pg/mL and 95.67 ± 4.041 pg/mL, respectively, in response to TNF-α/IFN-γ. As shown in Figure 4, 3% HM-V strongly inhibited the TNF-α/IFN-γ-induced production of CCL17 and CCL22. In addition, 3% HM-V significantly inhibited IL-6 production and decreased production of other pro-inflammatory cytokines (IL-1β, IL-8, and TNF-α).

## 3. Discussion

In this study, the inhibitory effects of HM-V on chronic inflammatory skin conditions were investigated. First, HaCaTs were used to verify the mRNA expression regulation activity of CCL17 and CCL22, which are biomarkers of atopic dermatitis. In addition, the effects of HM-V on the regulation of pro-inflammatory cytokine mRNA expression were examined. The anti-inflammatory activity of HM-V was confirmed using a DNCB-induced dermatitis animal model. HM-V reduced the increased epidermal thickness due to DNCB. In addition, treatment with HM-V suppressed the infiltration of mast cells and eosinophils. Furthermore, HM-V reduced the serum pro-inflammatory cytokine levels, including CCL17 and CCL22, as well as the size of the spleen and lymph nodes.

Chronic skin inflammation refers to a general skin barrier disorder characterized by skin lesions (such as dry skin, itching, eczema, and erythema) and an immune hypersensitivity reaction caused by the infiltration of immune cells (such as lymphocytes, eosinophils, and mast cells) [21,22,23]. Previously reports, various types of chronic dermatitis, including atopic dermatitis, were shown predominantly caused by activation of Th2 cells. In contrast, Th1 cells are involved in the chronic phases of these skin disorders [24,25,26]. Chemokines have been reported to play pivotal roles in the exacerbation of symptoms. In particular, CCL17 and CCL22 bind to selective CCR4 receptors, thus affecting Th2 cell activation and propagating ongoing immune imbalance [27]. The CCR4 specific ligands, CCL17 and CCL22, induce persistent immune cell accumulation at skin inflammation sites. In prior studies, chemokines such as CCL17 and CCL22 were suggested as potential therapeutic targets for chronic skin inflammation, including atopic dermatitis. In the present study, HM-V inhibited the protein production of CCL17, CCL22, and pro-inflammatory cytokines (IL-6, IL-8, IL-1β, and TNF-α in a dose-dependent manner in TNF-α/IFN-γ-stimulated HaCaTs). In addition, HM-V inhibited CCL17 and CCL22 mRNA expression. The results indicate that HM-V may attenuate skin inflammatory symptoms by downregulating CCL17 and CCL22 production in human keratinocytes.

Mouse animal models of topically treated DNCB that result in contact dermatitis are test methods for identifying chronic skin inflammation [28]. Increasing epidermal thickness and eosinophil and mast cell infiltration are major features of chronic skin inflammation such as atopic dermatitis [29,30]. Here, we confirmed that changes in the characteristics of skin inflammation were observed following HM-V treatment. Eosinophil and mast cell infiltration, and increasing epidermal thickness, were significantly inhibited in mice treated with 3% HM-V for 4 weeks. Furthermore, HM-V inhibited the production of pro-inflammatory cytokines including CCL17 and CCL22. In addition, HM-V treatment reduced the spleen and lymphocyte size.

HM-V is composed of various Asian medicinal herb extracts. One of the most important components is *Portulaca oleracea* L. extract, which contains a variety of flavonoids, such as kaempferol, apigenin, quercetin, and genistein, which reportedly has excellent anti-inflammatory, anti-oxidant, and anti-cancer activities. Also, according to recent research results, atopic dermatitis treatment activity was reported by inhibiting T-cell activation as a result of the regulation of phosphorylation of JNK in the study of kaempferol oral administration in mice. [31,32,33,34,35,36]. *Sophora flavescens* Aiton extract also reduces cytokine production via inhibition of NF-κB signaling in human mast cells [37]. It is also known that flavonoids such as 8-prenylkamperol, kushenol X, and norkurinone, which are major components from *Sophora flavescens* Aiton root extract, bind with estrogen receptors to exhibit physiological activity [38]. Chelidonine, an active ingredient of *Chelidonium majus* var. *asiaticum*, was shown to strongly inhibit the production of LPS-induced inflammatory mediators in mouse macrophage RAW264.7 cells [39]. Maybe, the active ingredients contained in the various plant extracts such as kaempferol, quercetin, and chelidonine that make up HM-V can be assumed to be the cause of the strong anti-inflammatory activity of HM-V. However, we believe that the analysis of the active ingredients contained in HM-V should be conducted to determine the causal relationship. In the present study, a new complex Asian medicinal herb extracts mixture, HM-V, was made using a traditional recipe and its activities were investigated. The results showed that HM-V inhibited the production of CCL17 and CCL22. In addition, HM-V exerted anti-inflammatory effects against DNCB-induced atopic symptoms in mice. HM-V suppressed the expression of pro-inflammatory cytokine genes in TNF-α/IFN-γ-stimulated HaCaTs. In summary, HM-V is a novel medicinal composite herb mixture with potent anti-inflammatory effects in DNCB-induced animal models of chronic skin inflammation. The results demonstrated the efficacy of Asian medicinal extract that has been used in Asia for many years, both in vitro and in vivo. The results indicate new possibilities for natural drug development in the treatment of chronic skin disease.

## 4. Materials and Methods

### 4.1. Medicinal Herb Extract Formulation

The solution A was prepared as follows: equal amounts (15 g dry weight) of 15 Korean traditional Asian medicinal ingredients were extracted using 60% butylene glycol solution for 72 h. The solution B was obtained by fermentation of methylpropanediol with Militaris cordyceps strains that were filtered before use. Gypsum and alum were mixed with purified water, followed by leaching and filtration to prepare solution C. The solution A (30%), solution B (10%), solution C (20.051%), colloidal silver (2%), and purified water (37.949%) were added to prepare the HM-V (100%) solution. In the present study, HM-V was composed of the following 15 herb extract mixtures: *Portulaca oleracea* L., *Sophora flavescens* Aiton, *Lonicera japonica, Chelidonium majus* var. *asiaticum*, *Dictamnus dasycarpus* Turcz, *Eriobotrya japonica* (Thunb.) Lindl, Heartleaf Houttuynia, *Lithospermum officinale*, *Viola mandshurica*, *Sanguisorba officinalis* L., *Gentiana scabra*, *Artemisia apiacea* Hance, *Taraxacum platycarpum* H. Dahlstedt, *Leonurus japonicus Houtt.*, and *Coptis japonica* Makino. The mixture was dissolved in water and diluted with cell culture media for in vitro assays.

### 4.2. Cell Culture and Viability Assay

HaCaTs were cultured in Dulbecco’s modified Eagle medium (Welgene, Seoul, Korea) with 10% fetal bovine serum (FBS), 2 mM glutamine, and 100 units/mL antibiotics (Gibco BRL, Rockville, MD, USA). The cells were incubated at 37 °C in a humidified atmosphere of 5% (*v*/*v*) air/CO_2_. The HaCaTs were inoculated into 96-well culture plates at 5 × 10^3^ cells/well and incubated for 18 h. After cell attachment and stabilization, the cell culture medium was replaced with fresh medium without growth factors. The incubated cells were treated with or without indicated concentrations of HM-V in serum-free medium for 24 h. Next, 100 μg/mL of 3-(4,5-dimetnythiazol-2-yl)-2,5-diphenyl-thetazolium bromide (MTT) was added to the treated and non-treated cells for 1 h. Purple formazan crystals were dissolved in 200 μL DMSO solution and the absorbance was measured at 560 nm using a multi-plate reader. The analyses were repeated three times. The results were expressed as means of three independent experiments.

### 4.3. Animals and Maintenance

The animal experiments were approved by the Institutional Animal Care and Use Committee at Kyung Hee University (KHGASP-20-172). Male BALB/c nude mice (6 weeks of age) were obtained from Orient Bio. Inc. (Sungnam, Korea). All mice were cared for at the animal facility of Kyung Hee University (Yongin, Korea). The mice were housed in a controlled environment with a 12-h light/12-h dark cycles and free access to food and water. The room temperature was maintained at 22 ± 1 °C with 50 ± 10% humidity. DNCB (Sigma-Aldrich, St. Louis, MO, USA) was dissolved in a working solution (3:1 acetone: olive oil) and used as a sensitizer to induce atopic dermatitis-like skin lesions in mice. The mice were divided into three groups with six or seven mice per group as follows: vehicle, DNCB, and DNCB plus topical treatments with 3% HM-V. As shown in Figure 2, the nude mice were treated for 1 week with 1% DNCB (100 μL), that caused contact dermatitis. Next, 3% HM-V solution in PBS (100 μL) was administered daily for 6 weeks and 0.5% DNCB was administered once every 2 days for the same period.

### 4.4. RT-PCR

The HaCaTs (1 × 10^6^ cells/well) were cultured in plates (60 mm/6-well plate) and treated with or without TNF-α/IFN-γ (10 ng/mL). The cells were incubated with indicated concentrations of HM-V for 24 h. Total RNA was isolated from HaCaTs using the TRIzol reagent kit (Invitrogen, Carlsbad, CA, USA) according to the manufacturer’s protocol. Common experimental methods were performed using M-MuLV reverse transcriptase (Fermentas Life Science, Burlington, Canada) to obtain cDNA from total RNA (2 μg). The obtained cDNA was used to amplify target mRNAs with gene-specific primers using the AccuPower PCRpre mix kit (Bioneer, Daejeon, Korea).

### 4.5. Enzyme-Linked Immunosorbent Assay (ELISA)

Treated HaCaTs were cultured under the same conditions as for the RT-PCR experiment, the culture medium collected, and the protein concentrations determined. The quantification of all proteins including, CCL17, CCL22, IL-1β, IL-6, IL-8, and TNF-α, were confirmed using an enzyme-linked immunosorbent assay (ELISA) kit (R&D Systems, Minneapolis, MN, USA) according to the manufacturer’s protocol.

### 4.6. Histological Analysis

Tissue was taken from the dorsal area of treated mice. The tissue was fixed in 3.7% formalin and then embedded in paraffin. The embedded tissues were sliced into 4.5-μM-thick sections and stained with hematoxylin/eosin (H&E) or Toluidine Blue O, according to the manufacturer’s protocol (Santa Cruz Biotechnologies, Santa Cruz, CA, USA). The epidermal thickness and invasiveness of the mast cells were observed using light microscopy.

### 4.7. Statistical Analysis

The data were expressed as means ± standard deviation (SD). The Student’s *t*-test was used to compare the two groups. Multiple group comparisons were performed using one-way analysis of variance (ANOVA). All experiments were performed in triplicate and repeated at least three times.

## Data Availability

The data presented in this study are available in this article.
